# Spiegelzymes: Sequence Specific Hydrolysis of L-RNA with Mirror Image Hammerhead Ribozymes and DNAzymes

**DOI:** 10.1371/journal.pone.0054741

**Published:** 2013-01-30

**Authors:** Eliza Wyszko, Maciej Szymański, Heinz Zeichhardt, Florian Müller, Jan Barciszewski, Volker A. Erdmann

**Affiliations:** 1 Institute of Bioorganic Chemistry of the Polish Academy of Sciences, Poznan, Poland; 2 Charite - University Medicine Berlin, Campus Benjamin Franklin, Institute for Virology, Berlin, Germany; 3 Pentafolium-Soft, Rosengarten, Germany; 4 Institute for Chemistry and Biochemistry, Free University Berlin, Berlin, Germany; Institute of Enzymology of the Hungarian Academy of Science, Hungary

## Abstract

In this manuscript we describe for the first time mirror image catalytic nucleic acids (Spiegelzymes), which hydrolyze sequence specifically L-ribonucleic acid molecules. The mirror image nucleic acid ribozymes designed are based upon the known hammerhead ribozyme and DNAzyme structures that contain L-ribose or L-deoxyribose instead of the naturally occurring D-ribose or D-deoxyribose, respectively. Both Spiegelzymes show similar hydrolytic activities with the same L-RNA target molecules and they also exhibit extra ordinary stabilities when tested with three different human sera. In this respect they are very similar to Spiegelmers (mirror image aptamers), which we had previously developed and for which it has been shown that they are non-toxic and non-immunogenic. Since we are also able to demonstrate that the hammerhead and DNAzyme Spiegelzymes can also hydrolyze mirror image oligonucleotide sequences, like they occur in Spiegelmers, *in vivo*, it seems reasonable to assume that Spiegelzymes may in principle be used as an antidote against Spiegelmers. Since the Spiegelzymes contain the same building blocks as the Spiegelmers, it can be expected that they will have similar favorable biological characteristics concerning toxicity and immunogenety. In trying to understand the mechanism of action of the Spiegelzymes described in this study, we have initiated for the first time a model building system with L-nucleic acids. The models for L-hammerhead ribozyme and L-DNAzyme interaction with the same L-RNA target will be presented.

## Introduction

Within the last 30 years it became apparent that natural nucleic acids may possess, besides their traditionally known functions in gene expression, an unforeseen structural and functional repertoire. This could range from enzymatic to high affinity binding functions. *In vitro* selection techniques were developed to produce high affinity nucleic acids, called aptamers, and also different types of ribozymes and DNAzymes. Thus, it was established that RNA and DNA molecules can be selected to carry out functions that were previously solely attributed to proteins [1].

Nucleic acid aptamers are single stranded DNA or RNA oligonucleotides, which are capable of adopting structures that bind any target imaginable with very high specificities and affinities. Thus, the binding properties of RNA and DNA aptamers can make them to excellent alternatives to antibodies [1], [2]. However, the use of aptamers is severely limited by the susceptibility of nucleic acids, and here especially of RNAs, to the hydrolysis by ubiquitous nucleases [3], [4]. The problem of the instability of RNA molecules can partially be overcome by chemical modifications [5], [6]. Another very promising possibility of obtaining very stable RNA aptamers has been previously been demonstrated by us with the development of Spiegelmers, which are mirror image nucleic acid aptamers [3], [4]. These Spiegelmers carry instead of the natural occurring D-ribose, L-ribose, and they are therefore resistant to natural occurring nucleases. The resistance of Spiegelmers against nucleases has previously been reported [3], [4], [6] and will also be shown in this communication.

Spiegelmers can be generated against a wide range of targets, since their development makes use of the methods of molecular evolution [6]–[10]. So far it has been possible to obtain Spiegelmers binding small molecules, such as D-adenosine [3] and L-arginine [4], or pharmaceutically relevant targets including vasopressin, gonadotropin releasing hormone, ghrelin, substance P, calcitonin gene-related peptide, pain-related neuropeptide nociceptin and relevant proteins, and *Staphylococcus* enterotoxin [7]. On the basis of our current knowledge it can be assumed that Spiegelmers can be developed for the same number of targets, as D-nucleic acid aptamers [3], [4]–[14]. The pharmacological properties of Spiegelmers are very favorable. No negative immunogenic response or toxicity has been detected so far [7]. Also, up to now, no adverse side effects have been reported for Spiegelmers, even when applied in relatively high doses [12], [13]. Spiegelmers have found a number of interesting applications in biotechnology, and recently have even been entered into their first and second clinical testing phases as potential pharmaceutical drugs [7], [8], [14].

In this communication we describe for the first time mirror image catalytic nucleic acids, i.e., L-RNA hammerhead ribozymes and L-DNAzymes, constructed on the basis of the known D-RNA hammerhead ribozyme and D-DNAzyme [15]–[24] structures. Because of their mirror image nature to the natural hammerhead ribozyme and DNAzyme, we named this class of nucleic acid enzymes Spiegelzymes (Spiegel stands in German for mirror). In this study we will show that left handed L-RNA ribozymes and L-DNAzymes are able to induce sequence specific and efficient hydrolysis of their homochiral RNA targets under *in vitro* and *in vivo* conditions. Thus, the Spiegelzymes fulfill a very essential and basic prerequisite as potential functional antidotes for Spiegelmers.

## Materials and Methods

### RNA and DNA Synthesis

All RNA and DNA oligonucleotides were chemically synthesized by ChemGenes (USA) or by IBA (Germany). The D-RNA1 substrate of 13 nucleotide length had the following sequence: 5′-A_1_CAGUCGGUCGCC_13_-3′. For the L-RNA1 substrate the same nucleotide sequence was used, except that the D-nucleotides were replaced by the corresponding L-nucleotides. Both target forms of RNA1 were labeled with fluorescein at their 5′ ends.

The D-RNA hammerhead ribozyme used for the D-RNA1 target had the following sequence 5′-G_1_GCGACCCUGAUGAGGCCGAAAGGCCGAAACUGU_34_-3′, while the L-RNA hammerhead ribozyme (Spiegelzyme) designed for the L-RNA1 target had the same sequence with the exception that L-nucleotides, instead of the D-nucleotides, were used in the sequence of the Spiegelzyme.

The 14 nt sequences of the D-RNA2 and L-RNA2 targets were the 5′-C_1_UUCAAGUCCGCCA_14_-3′. The difference between both targets was in that the D-RNA2 consisted of D-nucleotides and the L-RNA2 target of L-nucleotides. The L-RNA2 target actually corresponds with its sequence to a fragment of the green fluorescent protein (GFP) mRNA (positions 252 through 265). Both target forms of the RNA2 were labeled with fluorescein at their 5′ ends. The hammerhead ribozyme and hammerhead Spiegelzyme (34 nt) specific for RNA2 had the following sequence:

5′-U_1_GGCGCUGAUGAGGCCGAAAGGCCGAAACUUGA_33_-3′. The difference being that the hammerhead Spiegelzyme consisted of the L-form of the nucleotides.

The two different DNA1 substrates used were the 5′ fluorescein labeled D- and L- forms with the sequence 5′-A_1_CAGTCGGTCGCC_13_-3′. Thus, the sequences corresponded in principle to the target RNA1 sequence.

The chemically synthesized D-DNAzyme and L-DNAzyme were 27 nucleotides in length with the following sequence 5′-G_1_GCGGAGGCTAGCTACAACGATTGAAG_27_-3′ in the D- and L-form respectively.

### Target D-RNA1 Hydrolysis with D-Hammerhead Ribozyme and Target L-RNA1 Hydrolysis with L-Hammerhead Ribozyme (Spiegelzyme)

The D-RNA1 target was hydrolyzed with the D-hammerhead ribozyme under the following conditions: 0.2 µM 5′ fluorescein labeled D-RNA was incubated in 10 µl 50 mM Tris-HCl buffer, pH 7.5, at 37°C in the presence of 1–10 mM MgCl_2,_ at an ribozyme concentration of 2 µM, and for 2 h unless otherwise indicated in the legends of the figures. Prior to the reaction the target D-RNA1 and D-hammerhead ribozymes were denatured for 2 min at 73°C and cooled to the reaction temperature of 25°C at 1°C per min in a heating block.

The hydrolysis of the L-RNA1 target with the L-hammerhead ribozyme was carried out under identical conditions as described for the D-RNA1 hydrolysis by the D-hammerhead ribozyme.

### Target D-RNA2 Hydrolysis with D-DNAzyme and Target L-RNA2 Hydrolysis with L-DNAzyme (Spiegelzyme)

The activity of D-DNAzyme was analyzed in cleavage reactions, in which 0.2 µM of 5′ fluorescein labeled D-RNA2 was incubated in 10 µl of a reaction mixture containing 50 mM Tris-HCl buffer, pH 7.5, 10 mM MgCl_2_ at 37^o^ C for 3 h in the presence of 0.02–10 µM of D-DNAzyme. The enzyme: substrate ratio was 10∶1. Prior to the reaction, the substrate D-RNA2 and D-DNAzyme were denatured for 2 min at 73^o^C and cooled to the temperature of 25^o^C (1°C/min) in a heating block.

The hydrolysis of the L-RNA target with the L-DNAzyme was carried out as described for the D-RNA2 target hydrolysis by the D-DNAzyme, except that the chemically synthesized target and DNAzyme consisted of L-nucleotides.

### Stability Studies of L- and D-Hammerhead Ribozymes and L- and D-DNAzymes in Different Human Sera

Three different sera preparations where used: two sera were purchased from Sigma in 1996 and 2009 and one serum was derived from whole blood of a healthy blood donor, who signed the “Agreement for blood donation (Einwilligung zur Blutspende)” incl. a detailed questionnaire which German Red Cross Blood Donor Service East uses for interviewing each blood donor before blood collection.

Blood was collected without anticoagulant and clotting was allowed over night at room temperature. The serum tested was prepared from whole blood of a healthy blood donor who donated blood at German Red Cross Blood Donor Service East (DRK-Blutspendedienst Ost gemeinnützige GmbH). Serum was prepared by centrifugation for 30 min at 3000 rpm in a Heraeus Megafuge 3 or at 4°C and stored in aliquots at −25°C. Serum was tested negative for HIV and hepatitis B and C by routine serological tests.

### Stability Studies of L-RNA2 with T1, V1, S1, and T2 Nucleases

The stability of 0.2 µM 5′ labeled fluorescein labeled L-RNA1 substrate was studied with T1, V1, S1, and T2 nucleases. The L-RNA 14-mer was incubated in a 10 µl reaction volume with the ribonucleases: 10 U T1 ribonuclease (Ambion) was incubated with the L-RNA1 substrate in a buffer containing 20 mM sodium citrate (pH 5.0), 7 M urea and 1 mM EDTA, at 55°C for 20 minutes. RNase V1 (Ambion) digestion was carried out by using 10 U of the ribonuclease for 30 min at 25°C in a buffer containing 50 mM Tris-HCl pH 7.2, 100 mM NaCl and 10 mM MgCl_2._ The RNase S1 (Promega) digestion was performed by incubating 5 U of the enzyme for 45 min at 37°C in a reaction buffer consisting of 50 mM sodium acetate (pH 4.5), 28 mM NaCl and 4.5 mM ZnSO_4_. The RNase T2 (Promega) digestion was performed by incubating 2 U enzyme with the L-RNA2 sequence for 15 min at 37°C. The reaction buffer consisted of 50 mM sodium acetate pH 4.5, 28 mM NaCl and 4.5 mM ZnSO_4_.

The RNA sequence ladders were obtained by alkaline hydrolysis. The incubation was at 95°C for 2 min in 10 µl of the reaction mixture containing 50 mM NaOH, 1 mM EDTA and 4 µg of crude tRNA (Serva) and 4 µg of 5′- fluorescein labeled L-RNA2.

### Gel Electrophoresis of Enzymatic Reaction Mixtures

Unless otherwise stated the reaction products were separated by 20% polyacrylamide gels containing 7 M urea in 0.09 M Tris-borate buffer, pH 8.3, and analyzed by a Fuji Film FLA 5100 phosphoimager by using the manufacturer’s software. The polyacrylamide gels were run at room temperature for four to six hours using bromophenyl blue as a marker.

## Results and Discussion

There were several reasons, which prompted us to try to develop the Spiegelzymes described in this study. After we had originally developed the mirror image aptamers called Spiegelmers [3], [4], it was of interest to see, if we could also develop mirror image nucleic acid enzymes (Spiegelzymes), cleaving sequence specifically other mirror image nucleic acid targets. On the other hand, we had also previously observed by x-ray analysis of crystals grown under microgravity conditions that the D- and L-forms of RNA molecules differ, not only in their chirality, but that there were also some differences in which divalent cations and water molecules could interact with the mirror image molecule at certain positions [25], [26]. This observation could also be very interesting, when the biological activities of the D-form ribozymes were compared with the Spiegelzymes. In addition, employing Raman spectroscopy analysis on the enantiomeric molecules, we could find out that the natural occurring D-RNA molecules exhibit a preferential stabilization over the L-RNA forms [27]. Thus, it would also be of interest to see if these structural differences could also be correlated to functional differences. Finally, we were also motivated to perform these experiments in order to find out, if we could develop mirror image nucleic acid enzymes, which would hydrolyze sequence specifically L-nucleic acid sequences. If this were the case, it would then indicate to us that the construction of highly specific antidotes towards L-nucleic acids, like Spiegelmers, would be worth to pursue [7], [8], [14].

### Concept to Study L-Hammerhead Ribozyme Activities

**Figure 1:**
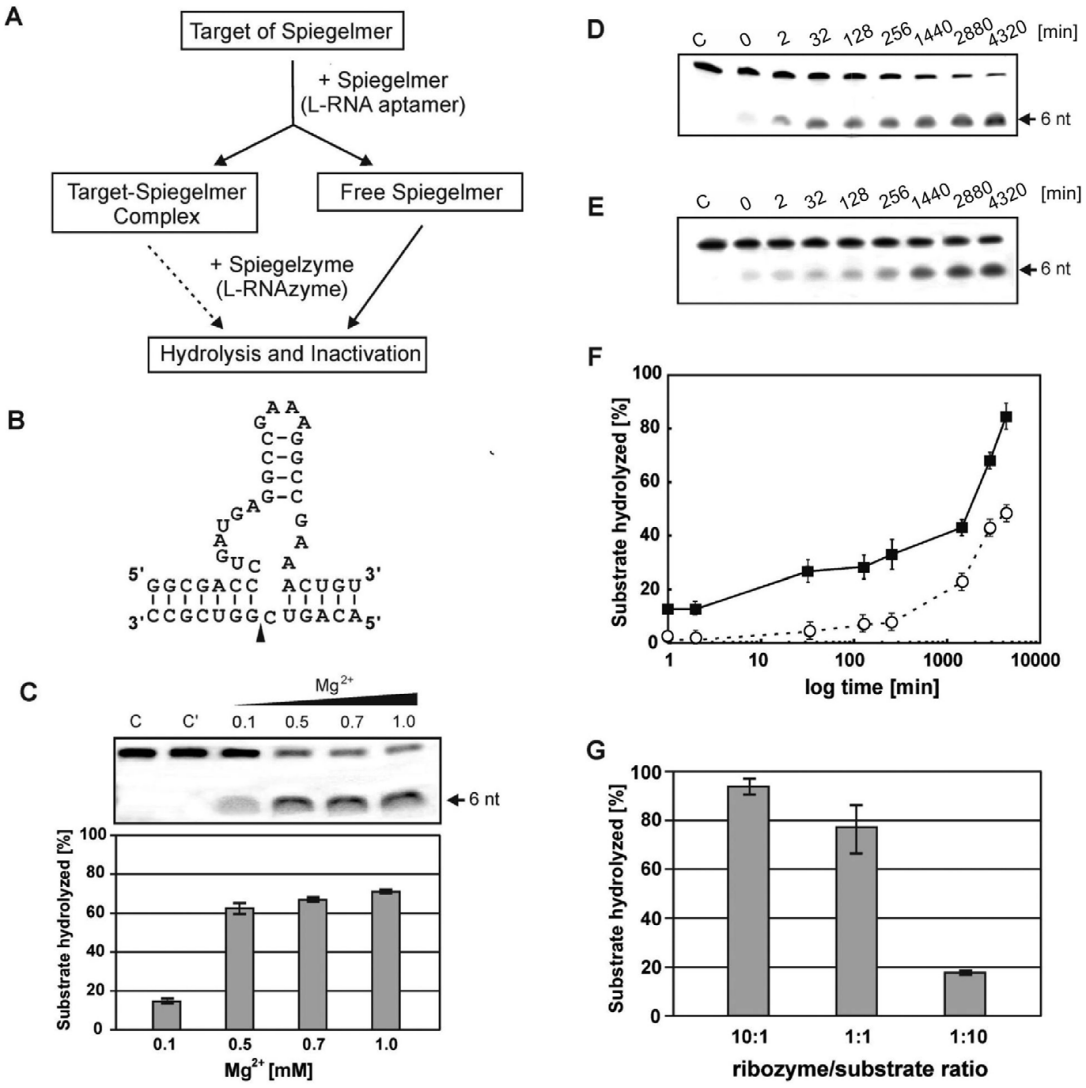
L-RNA1 Hydrolysis with an L-Hammerhead Ribozyme. (A) Scheme for the potential inactivation of Spiegelmers (L-RNAs) by Spiegelzymes (L-hammerhead ribozyme). (B) The L-RNA1 and the L-hammerhead ribozyme are presented as the nucleotide sequence specific homochiral complex. The cleavage site (GUC) in the target L-RNA1 is indicated by an arrow. (C) Analysis of Spiegelmer hydrolysis at 0.1, 0.5, 0.7 and 1 mM MgCl_2_. Control reactions were carried out with target L-RNA1 alone in buffer and 1 mM MgCl_2_ (lane C) or with target L-RNA1 and L-hammerhead ribozyme in buffer without Mg^2+^ (lane C’). (D, E) Time dependent cleavage of the L-RNA1 target with the L-hammerhead ribozyme (Spiegelzyme). The hydrolysis of 0.2 µM target fluorescein labeled L-RNA1, with 2 µM or 0.02 µM Spiegelzyme for single (D) and multiple (E) turnover reactions. The reactions were carried out in 50 mM Tris-HCl, pH 7.5, buffer containing 1 mM MgCl_2_ at 37°C in total volume of 200 µl. Lanes 1–8 show the hydrolysis product (6 nt long) after 0, 2, 32, 128 and 256 min, and 24, 48 and 72 h, respectively. (F) A plot of single (squares, solid line) and multiple (open circles, broken line) turnover reactions. (G) Hydrolysis of the L-RNA1 target with the hammerhead Spiegelzyme in human serum (Sigma 2009) at different Spiegelzyme to substrate ratios (10∶1, 1∶1, 1∶10). The reactions were carried out at 37°C for 2 h. All reaction products were separated by 20% polyacrylamide gel electrophoresis (PAGE) with 7 M urea (other details see Materials and Methods) and evaluated by fluorescence measurement with Fuju Film FLA 5100 phosphoimager (F).

The concept of this approach is shown in Figure 1A and the sequences involved have been demonstrated is shown in Figure 1B. In this cleavage reaction a hammerhead ribozyme was constructed in its mirror image form, so that we had an L-RNA hammerhead, which we also call a hammerhead Spiegelzyme, cleaving an L-RNA target (L-RNA1). Thus, we engineered a 34 nucleotide long L-RNA hammerhead ribozyme (Spiegelzyme) for the specific cleavage of a 13 nucleotide long substrate L-RNA1, containing an …NGUC↓N… sequence at the hydrolysis site (Fig. 1A, B). *In vitro* analysis demonstrated that the L-ribozyme can efficiently hydrolyze the fluorescein labeled substrate at the predicted site, producing a 6 nucleotide long fluorescently labeled product (Fig. 1C).

Studying the Mg^++^ dependence of the Spiegelzyme reaction, we observed that at 0, 0.1, 0.5, 0.7 and 1 mM MgCl_2_, 0, 14, 60, 65 and 75% of the target L-RNA1 was hydrolyzed (Fig. 1C). Higher magnesium concentrations of 5 and 10 mM (data not shown) did not improve the yield of the reaction shown in Fig. 1C for 1 mM MgCl_2_ at 37°C and 2 h reaction time.

To investigate the kinetic properties of the L-hammerhead ribozyme in more details, we analyzed the substrate hydrolysis at single (Fig. 1D, F) and multiple (Fig. 1E, F) turnover conditions, by using a 10-fold excess of L-ribozyme to L-substrate and L-substrate to L-ribozyme, respectively. A similar experiment was was performed with the D-hammerhead and D-RNA1 target (data not shown). As can be seen in the Fig. 1G the hydrolysis observed for the L-RNA1 target gave yields of 90 and 20% under these conditions.

The calculated K_obs_ for the L-hammerhead ribozyme were 0.014 and 0.024 min^−1^ at single and multiple turnover conditions. Similar experiments performed with a D-hammerhead ribozyme and a D-RNA target yielded K_obs_ of 0.015 and 0.011 min^−1^ at single and multiple turnover conditions (data not shown). Thus, we conclude that both enzymatic reactions exhibit very similar kinetic properties.

The Spiegelzyme also exhibited very promising catalytic properties under *in vitro* conditions with human sera, i.e., in an commercially purchased human serum (Sigma 2009), and it showed at L-hammerhead to L-RNA1 ratios of 10∶1, 1∶1 and 1∶10 very effective hydrolysis levels of 94, 77 and 18%, respectively (Fig. 1G). These results indicate that ionic and buffer conditions are sufficient for the Spiegelzyme to cleave the target L-RNA1 sequence specifically in human serum. Thus, the results suggest that the L-hammerhead ribozyme catalyzed cleavage of the L-RNA1 target could most likely also takes place in living cells.

### Can L-Hammerhead Ribozymes Cleave L-DNA Targets?

**Figure 2:**
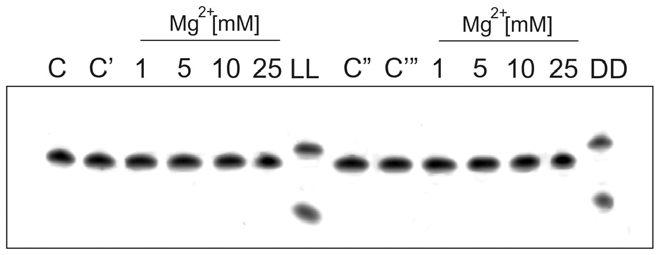
Hydrolysis of L-DNA1 by an L-Hammerhead Ribozyme and D-DNA1 by a D-Hammerhead Ribozyme at various Mg^++^ Concentrations. Left panel: Target L-DNA1 in buffer without MgCl_2_ (C), the same with 25 mM of MgCl_2_ (C’), Target L-DNA1 with a hammerhead Spiegelzyme at 1, 5, 10 and 25 mM MgCl_2_, LL control: L-RNA1 incubated with L-hammerhead. Right panel: Target D-DNA1 in buffer without (C’’) and with 25 mM MgCl_2_ (C’’’). Target D-DNA1 and hammerhead ribozyme at 1, 5, 10 and 25 mM MgCl_2_, DD control: D- RNA1 incubated with D-hammerhead ribozyme. Arrow identifies hydrolysis site as in Fig. 1B.

In another series of experiments we wanted to determine whether the L-hammerhead ribozyme used in this study would be able to hydrolyze an L-DNA1 substrate, and if a D-hammerhead ribozyme would be able to hydrolyze a D-DNA1 substrate. As expected no activities were observed in MgCl_2_ range between 1 and 25 mM (Fig. 2). The other experiments indicated in the Figure 2 with LL and DD represented the homochiral control experiments in which the L-hammerhead ribozyme hydrolyzed as expected the target L-RNA1 and the D-hammerhead ribozyme as also expected the target D-RNA1. Thus, it can be concluded that an L-hammerhead ribozyme cannot cleave an L-DNA sequence.

### Stability Studies of L-Hammerhead Ribozymes and L-RNA Oligonucleotides in Human Blood Sera and Specific Ribonucleases

**Figure 3:**
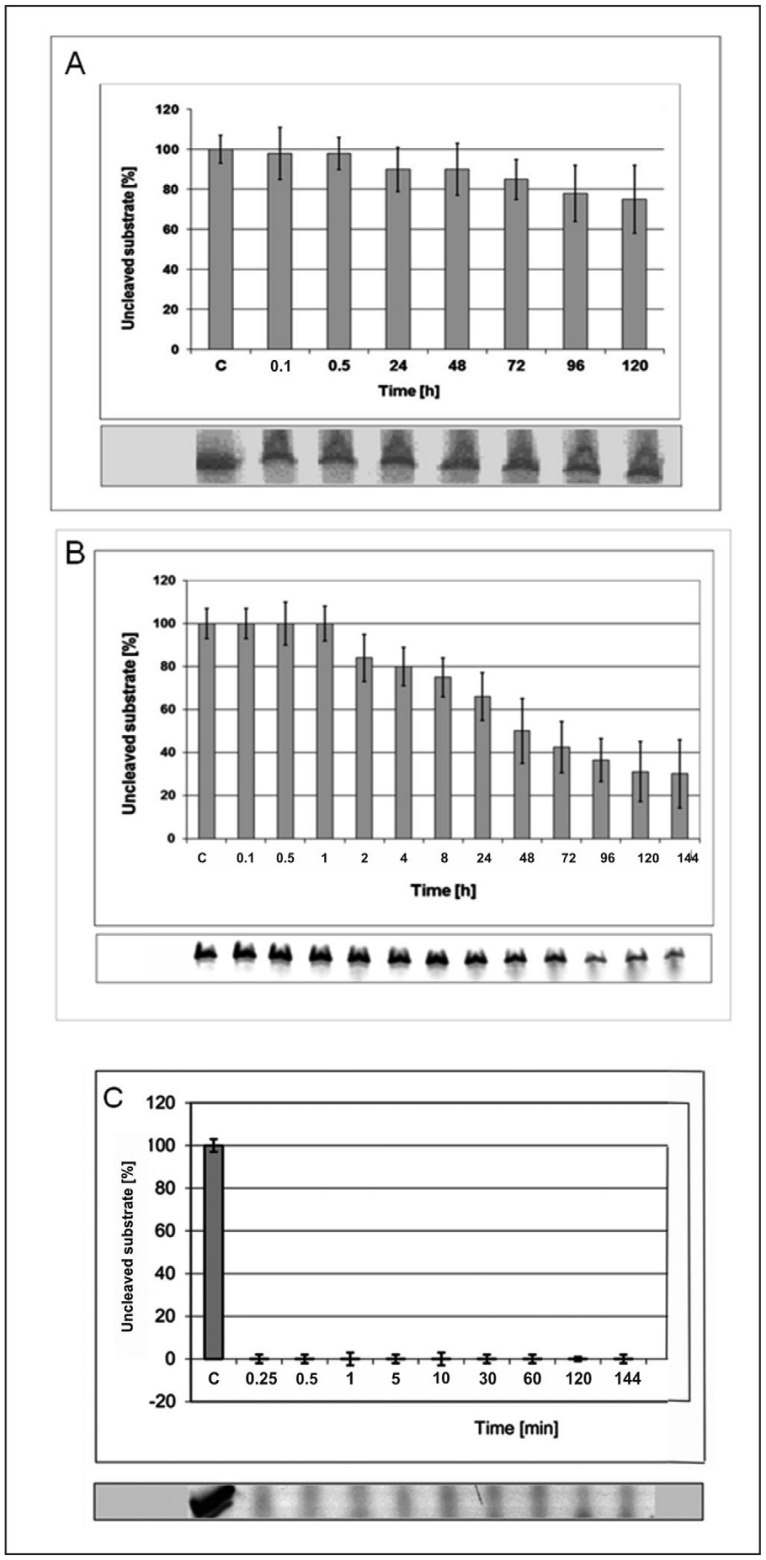
Hammerhead Spiegelzyme (34-mer) Stability in Human Blood Sera. (A) Incubation at 37°C for 0.25–120 h (6 days) in a human blood serum purchased from Sigma [4]. (B) Incubation at 37°C for 0.25–144 h in serum derived from a whole blood of a healthy blood donor. Blood was collected without anticoagulant and clotting was allowed over night at room temperature. Serum was prepared by centrifugation for 30 min at 3000 rpm in a Heraeus Megafuge 3 or at 4°C und stored in aliquots at −25°C. Serum was tested negative for HIV and hepatitis B and C by routine serological tests. (C) Control-Hammerhead ribozyme (same sequence as the Spiegelzyme) was incubated in serum (as in A) at 37°C for 0.25–120 min. All diagrams show fractions of the intact L-RNA, or D-RNA at a given time point. C-control sample was incubated on ice over time period given.

In order to analyze the stabilities of the L- and D-hammerhead ribozymes in human serum the experiments shown in Figure 3 were performed. For this experiment two sera of different sources were used. One was purchased from Sigma in 2009 and it was, according to Sigma, similarly prepared as the one used in our earlier stability studies of Spiegelmers in 1996 [3], [4], and the second serum was a donated serum from a patient from the Charite Hospital Berlin (Fig. 3A and B). The experiments showed that the hammerhead Spiegelzymes are very stable in the two sera over a time period of 120 and 144 hrs respectively, and that the stabilities are similar than those observed for the Spiegelmers in the human serum from Sigma in 1996 [3], [4]. The superior stabilities of the mirror image hammerhead ribozymes becomes especially apparent from the control experiment, in which the hammerhead ribozyme (D-RNA) was incubated with the donated patients serum and which was already totally hydrolyzed at 1 min on ice (Fig. 3C).

**Figure 4:**
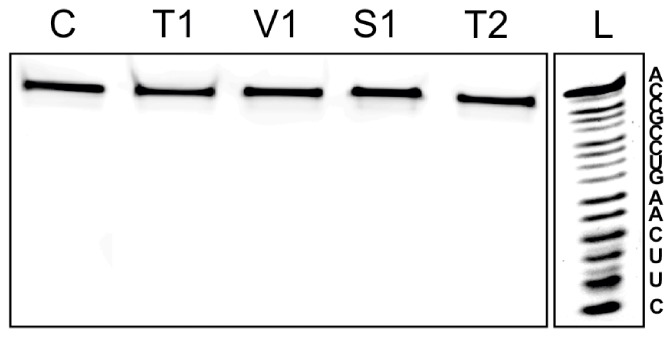
Hydrolysis Experiments of L-RNA2 with T1, V1, S1, and T2 Ribonucleases. Incubation conditions as described under Materials and Methods. L: alkaline L-RNA2 ladder. The nucleotide sequence of L-RNA2 is indicated. All nucleases were active against D-RNA (data not shown).

Although we could prove the stability of L-RNAs in human sera, it was also of interest to know if the L-RNAs are also resistant to isolated specific nucleases like T1, V1, S1, and T2. The results shown in Figure 4 clearly demonstrate that the target L-RNA1 is not hydrolyzed by any one of these ribonucleases. Thus, also with this experiment it is documented that the mirror image RNAs are of superior stability against all different types of nucleases. This finding would be one of the essential prerequisite if one would want to employ L-hammerhead ribozymes in cell culture, or animals or humans.

**Figure 5:**
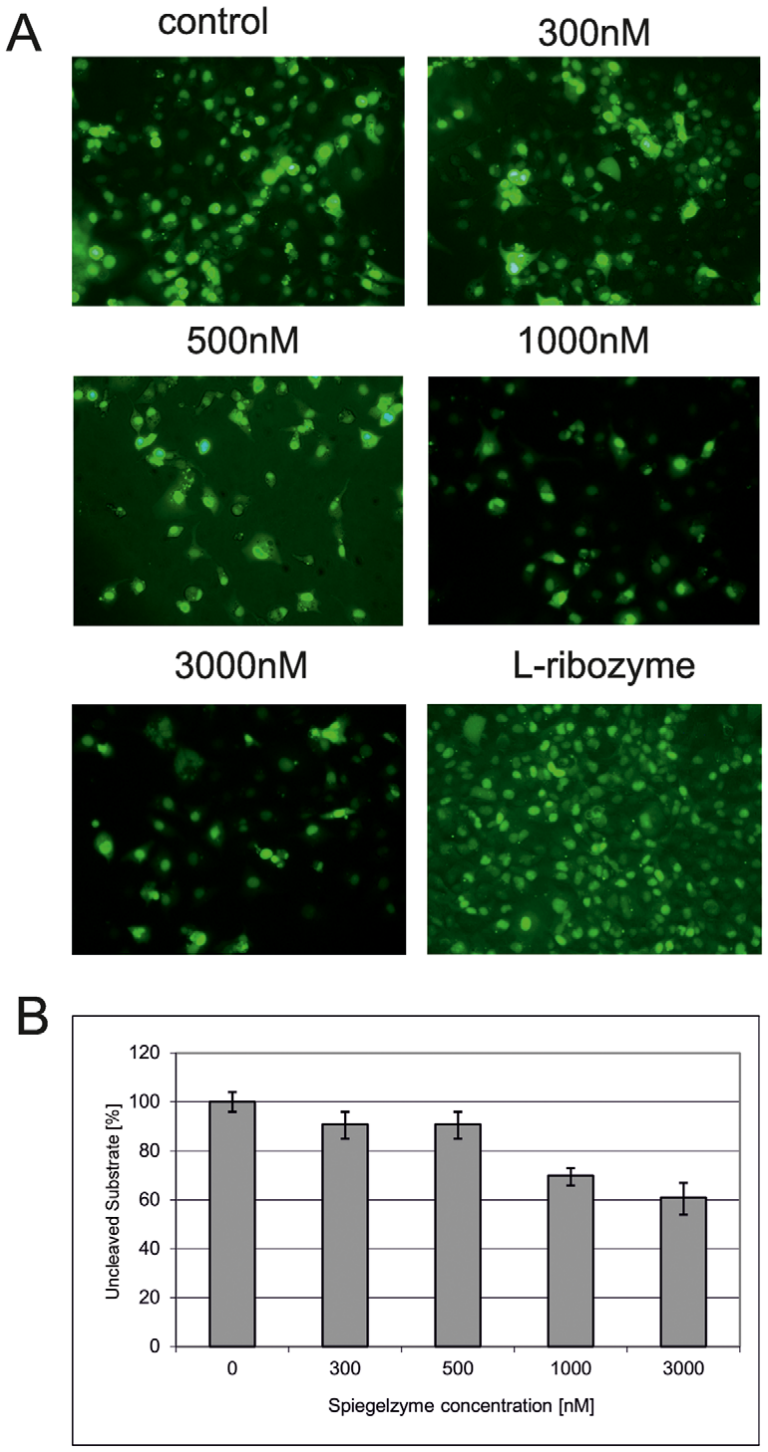
Hammerhead Spiegelzyme Activities in COS-7 cells. (A) Cells were transfected with 5′-fluorescein labeled L-RNA-1 substrates. Prior to the application of the HH Spiegelzyme, the cells were washed to remove the untransfected substrate from the medium. Then the cells were transfected with 300, 500, 1000 and 3000 nM of the Spiegelzyme. The control transfection was done only with the substrate. To check for the ability of the Spiegelzyme to cross the membrane, the cells were transfected with the fluorescein labeled Spiegelzyme (bottom row, right panel). The microscopic images were taken prior to RNA isolation after washing with PBS buffer. (B) The percentage of uncleaved substrate in the L-RNA-1 isolated from cells transfected with the Spiegelzyme. After incubation for 24 hours, L-RNA was isolated from harvested cells using TRIZOL (Ambion) according to the manufacturer’s protocol. L-RNA-1 was separated by 20% PAGE with 8 M urea and the amount of substrate was determined by the fluorescence intensity using Fuji Film FLA 5100 phosphoimager.

The hydrolytic activity of the Spiegelzyme was analyzed in the cells, transfected first with L-RNA substrate and the following day with the Spiegelzyme. A dose-dependent, 10–40% decrease of L-RNA isolated from the cells treated with the Spiegelzyme after 24 hours of incubation was observed (Fig. 5).

The above results show for the first time that mirror image catalytic nucleic acids (Spiegelzymes) can be constructed on the basis of a hammerhead ribozyme and that these Spiegelzymes are able to efficiently hydrolyze target L-RNA molecules and that this hydrolysis can also take place in human sera. Fulfilling these important prerequisites, it can be foreseen that Spiegelzymes may qualify for *in vivo* applications, as for example, as a Spiegelmer antidote [28], [29]. The development of such highly specific antidotes would be very desirable, especially, if one considers the fact that Spiegelmers, because of their stability, will have long pharmacological effects and that this would also be true for unforeseen side reactions. So far complementary oligonucleotides have been developed to neutralize unwanted side effects for aptamers by binding to them [29]–[31]. With the Spiegelzyme antidote potentials reported in this study, perfect antidotes would be at hand to neutralize the Spiegelmers, not only by complementary oligonucleotide binding, but also by sequence specific hydrolysis and thus by enzymatic inactivation.

### Construction of L-DNAzymes and their Biological Activities

Next we were interested to analyze the cleavage properties of another type of nucleic acid ribozyme, i.e. the DNAzyme, which is a catalytically active single-stranded DNA. DNAzymes recognize and hydrolyze RNA targets containing a …NG↓UN… sequence, in which N stands for anyone of the common four bases [32]. Advantages of the DNAzymes over hammerhead ribozymes are its easy chemical synthesis, broad target recognition properties, and a high catalytic turnover. Numerous application of these catalytic molecules have been tested, in particular as anticancer therapeutics [22], [33].

**Figure 6:**
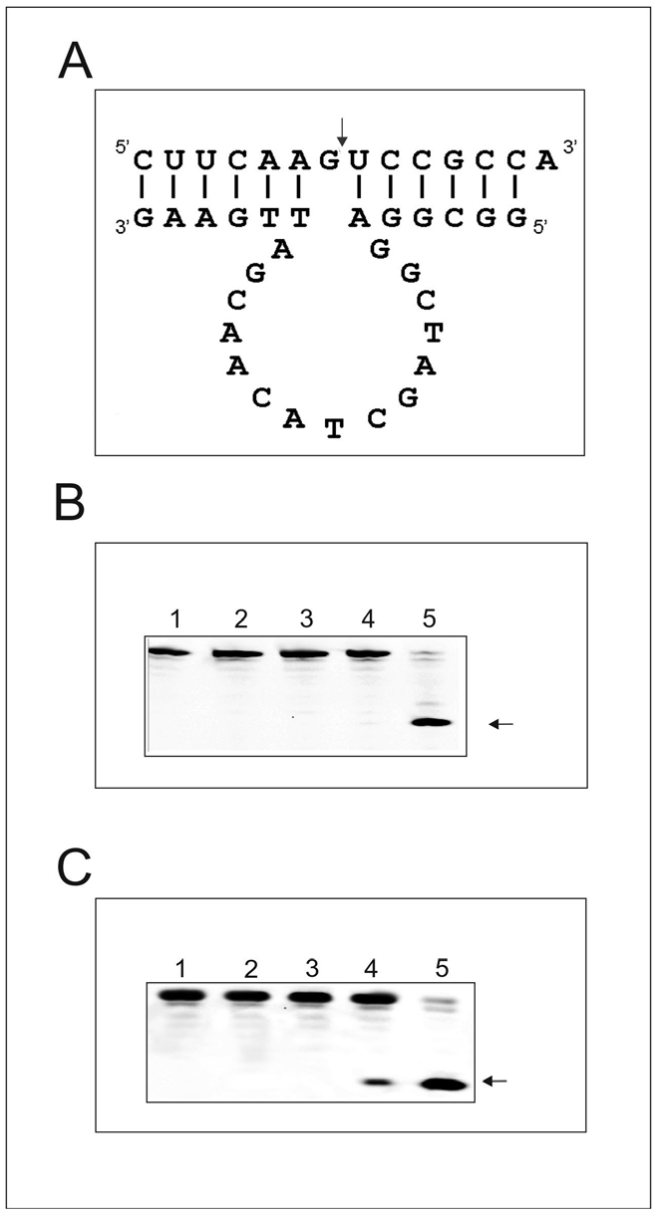
D- or L-RNA2 Hydrolysis by D- or L-DNAzyme. The general secondary structural model for the D- or L-DNAzyme 10/23 (A), in complex with a D- or L-RNA2 substrate (B). Hydrolysis of D-RNA2 with D-DNAzyme at 10 mM MgCl_2_. Lanes 1: D-RNA2 incubation in water, lane 2: D-RNA2 incubation in 50 mM Tris-HCl pH 7.5 buffer, lane 3: D-RNA2 incubation in the buffer containing in addition 10 mM MgCl_2_; lane 4: D-RNA2 hydrolysis with D-DNAzyme in buffer in the absence of MgCl_2_; lane 5: D-RNA2 hydrolysis with D-DNAzyme in buffer in the presence of 10 mM MgCl_2_ (C). L-RNA2 hydrolysis with L-DNAzyme at 10 mM MgCl_2_, analyzed with 20% PAGE with 7 M urea. Lanes 1: L-RNA2 incubation in water, lane 2: L-RNA2 incubation in 50 mM Tris-HCl pH 7.5 buffer, lane 3: L-RNA2 incubation in buffer containing in addition 10 mM MgCl_2_, lane 4: hydrolysis of L-RNA with L-DNAzyme in the absence of MgCl_2_; lane 5: hydrolysis of L-RNA substrate with L-DNAzyme in presence of 10 mM MgCl_2,_ All incubations were carried out for 3 hrs and analyzed with 20% PAGE with 7 M urea. Arrows show the specific cleavage site in the D- and L- RNA2 targets.

In the study reported here we designed an L-DNAzyme 10/23 based on L-deoxyribose and we checked its cleavage properties with an L-RNA2 target (Fig. 6A). The L-RNA2 target used corresponds in its sequence to positions 252 through 263 of the green fluorescent protein (GFP) mRNA. The results shown in Figure 6B and C demonstrate that the L-DNAzyme recognizes and hydrolyzes the L-RNA2 target and, as expected, that the normal D-form of the DNAzyme also hydrolyses the D-RNA2 target very efficiently.

### Enzymatic Activities of L-Hammerhead Ribozymes and L-DNAzymes at Different Ribozyme to Target Ratios

**Figure 7:**
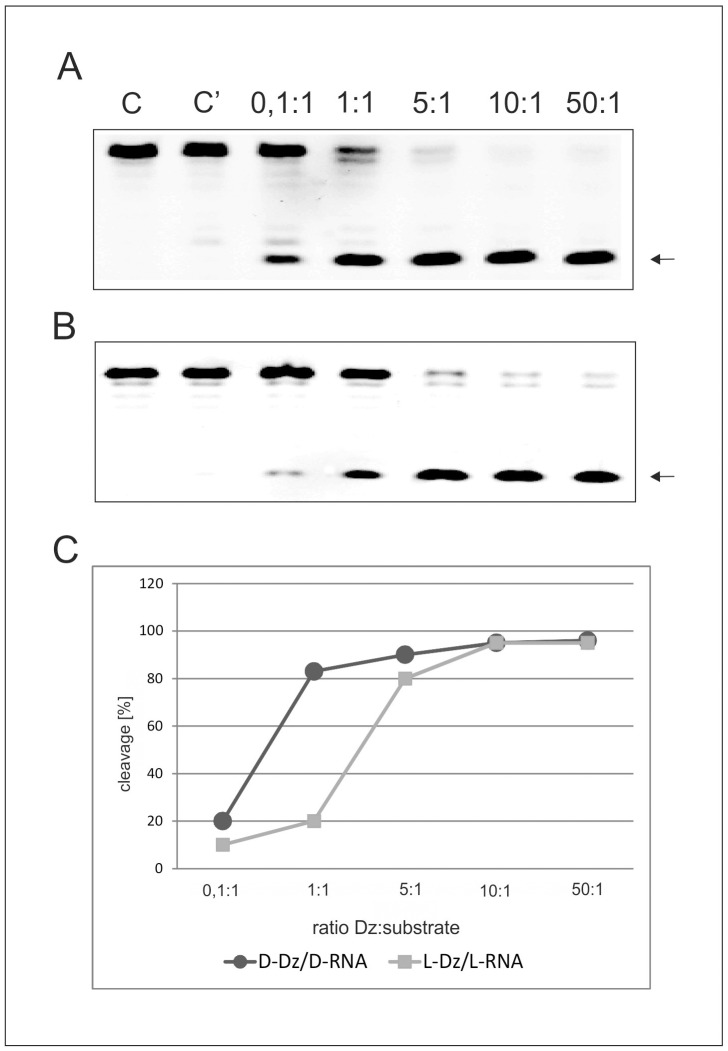
Hydrolysis of D- or L-RNA2 at different D- or L- DNAzyme ratios. Panel (A) D-DNAzyme hydrolysis of D-RNA2 at enzyme to substrate ratios as indicated in the lanes of the figure. Lane C: D-RNA2 incubated only in buffer, lane C’: D-RNA2 incubated in buffer with 10 mM MgCl_2_. Panel (B) L-DNAzyme hydrolysis of L-RNA2 at enzyme to substrate ratios as indicated in the lanes of the figure. Lane C: L-RNA2 incubated only in buffer, lane C’: L-RNA2 incubated in buffer with 10 mM MgCl_2_. (C) D-DNAzyme hydrolysis of D-RNA2 (black circles) at enzyme to substrate ratios as indicated in the figure and L-DNAzyme hydrolysis of L-RNA2 (gray squares) at ribozyme to substrate ratios shown in the panel. Incubation periods 3 h for all other conditions see Materials and Methods.

In the next series of experiments we compared the hydrolytic efficiencies of the D-DNAzyme with the D-RNA2 target (Fig. 7A) and the L-DNAzyme and the L-RNA2 target (Fig. 7B) at different ribozyme to substrate rations. As can be seen in Figure 6 the chosen ratios of enzyme to target ranged from 0,1∶ 1, 1∶1, 5∶1, 10∶1 to 50∶1. Under the reaction conditions of 10 mM MgCl_2_ and 3 hrs reaction time, a nearly 100% hydrolysis efficiency was reached for both D- and L-homochiral cases at an enzyme to substrate ration of 10∶1 (Fig. 7C), indicating for both ribozyme cases similar reaction efficiencies.

### Biological Stabilities of L-DNAzymes and D-DNAzymes in Different Human Sera

**Figure 8:**
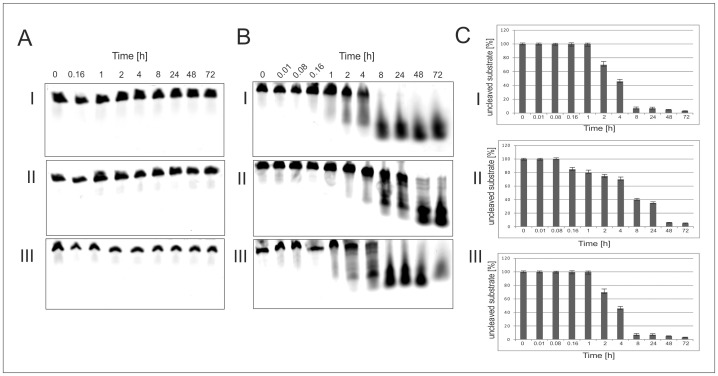
L-DNAzyme (27-mer) Stability in Different Human Sera. Panel A: L-DNAzyme (Spiegelzyme), Panel B. D-DNAzyme and Panel C: The % fraction of D-DNAzyme degraded at given times. Sera used in these experiments Sigma 2009 (I), Sigma 1996 (II) and serum isolated from a patient (III). The DNAzymes were incubated with the sera at 37**°**C for the times indicated in hrs in the panels. Other details see Materials and Methods.

Of interest to us was also the question, if the L-DNAzyme would show similar stability characteristics in different human sera, as the L-hammerhead ribozyme. As expected the results summarized in Fig. 8 demonstrate that the mirror image L-DNAzyme is very stable in the three human sera tested, showing no degradation over a time period of 72 h (Fig. 8A). Somewhat surprising is the observation that the D-DNAzyme showed, even after 4 h incubations, in the three different sera a significant resistant toward enzymatic degradation (Fig. 8B). This is in contrast to the D-hammerhead ribozyme, which showed total hydrolysis even when incubated at 0°C for 0 minutes (see Fig. 3C). These results most likely reflect to a certain degree the fact that RNA is in general much less stable than DNA in cellular fluids.

### Three Dimensional Model Building of L-Hammerhead Ribozyme and L-DNAzyme Interactions with an L-RNA Target

The data presented so far in this manuscript demonstrate for the first time, the construction of two mirror image enzymes, called Spiegelzymes, namely the L-hammerhead ribozyme and L-DNAzyme, which can sequence specifically recognize and hydrolyze homochiral target RNA molecules at low magnesium concentrations *in vitro*. These data show also that mirror image nucleic acids enzymes like L-RNA hammerheads and L-DNA DNAzymes are equally active as their corresponding enantiomers in homochiral reactions. Since some slight structural differences have been reported earlier from us on the basis of x-ray analysis of RNA crystals from D- and L-RNA molecules, and since these differences did also involved divalent cation and water interactions [26], [27], it becomes very intriguing to determine the mechanisms of catalysis of the mirror image enzymes studied in these experiments. Thus, we have initiated crystallization experiments involving the L-hammerhead ribozyme and L-DNAzyme complexes with their non-cleavable homochiral targets in order to determine their atomic structures. With these structures indications for the mechanisms of these reactions may be obtained and compared with those for the D-hammerhead ribozyme as proposed by Scott [20], [34].

**Figure 9:**
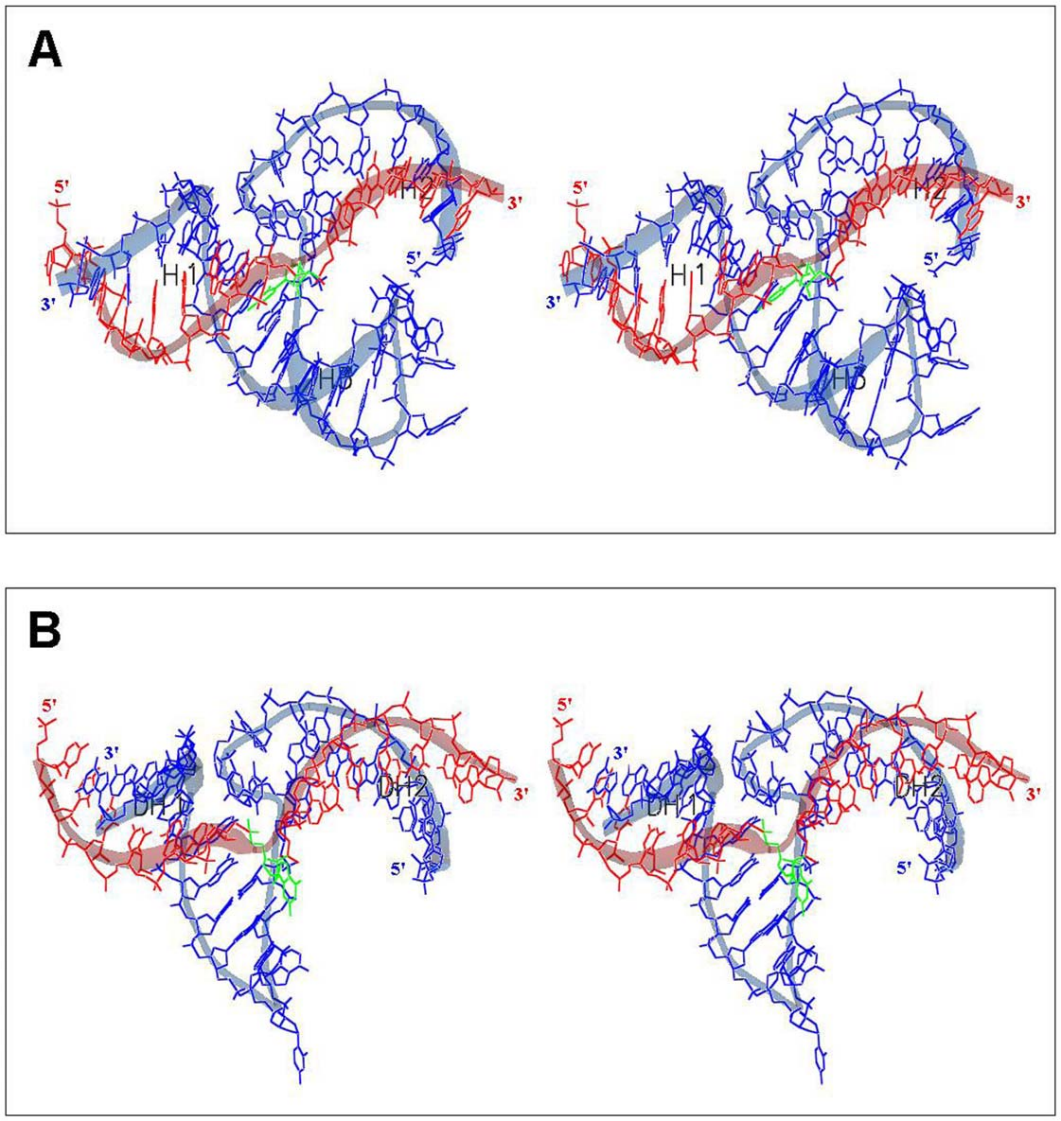
Atomic Models Proposed for the L-Hammerhead Ribozyme and the L-DNAzyme Interactions with their Target L-RNA2. (A) L-HHRz, 5′-U_1_GGCGCUGAUGAGGCCGAAAGGCCGAAACUUGA_33_-3' (shown in blue) with L-RNA2 target nucleotide sequence 5′-C_1_UUCAAGUCCGCCA_14_-3′ (shown in red) with the cleavage site at nucleotide C9 (shown in green), (B) L-DNAzyme 5′-G_1_GCGGAGGCTAGCTACAACGATTGAAG_27_-3′ (shown in blue) with L-RNA2 target nucleotide sequence 5′-C_1_UUCAAGUCCGCCA_14_-3′ (shown in red) with the cleavage site at nucleotide G7 (shown in green). See text for detail discussions of the models.

Nevertheless, since the atomic structures of our L-enzymes with their L-substrates are currently not known, it may be of interest to try to get an insight of the mechanism of action of theses Spiegelzymes by model building. The first attempt in this direction is shown in Fig. 9.

Figure 9A shows a stereo view of a wire-ribbon model of our L-RNA hammerhead ribozyme (shown in blue) in complex with the L-RNA2 target (shown in red). This preliminary model has been created by using the 2.2 Å structure (PDB-ID: 2GOZ) of the full-length catalytically active hammerhead ribozyme published [34]. This model has been created by first making a simple copy of the (D-form) highly conserved catalytic centre, which is incapable of forming canonical Watson–Crick base-pairs. Due to the pure copy of this catalytic centre all relevant nucleotides (G36 as well as A21 in the structure 2GOZ) lie close enough together, to ensure the described enzymatic hydrolysis at the cleavage-site nucleotide (OMC6 in structure 2GOZ and C9 in our model; shown in green in the Fig. 9A) of the substrate. After that, we built up the atomic models of three (D-RNA) double helices (H1 to H3) in a standard A-form by using the secondary structure shown in Fig. 1, but using the D-RNA2 instead of the D-RNA1 shown in that figure. In the case of double helix H3, the shape of the hairpin loop has been created automatically by the hairpin loop folding algorithm of our ERNA-3D Molecular Modeling Software (PENTAFOLIUM SOFT). Subsequently, we superimposed these three double helices over the three double helices (STEM-I to STEM-III) of the hammerhead ribozyme structure by fitting the bodies of the three helices as best as possible onto the given STEM helices. Due to the different nucleotide sequences between the hammerhead ribozyme (structure 2GOZ) and our proposed model, the hammerhead ribozyme so far has been used only as submittal shape for the spatial path of the nucleotide sequences. The double helices have been superimposed in such a way, that the docking points (the P- respectively the O3′-atoms) at the ends of each helix, which have to be close enough to both single strands of the catalytic centre, have been used as the starting points of the helices. In this way, we were able to connect the different helices with both single strands of the catalytic centre, without the need to change the spatial shape of the conserved parts of the hammerhead ribozyme structure. At last, the L-form of the molecule has simply been created by the negating of the z-components of the atomic coordinates [26], in this way the shown mirror image of the molecule could be created very easily.

In Figure 9B we are showing our attempt to construct a stereo view of a wire-ribbon model of an atomic model for the L-DNAzyme (shown in blue) in complex with same L-RNA2 target (shown in red) and with the cleavage-site at nucleotide G7 (shown in green). The preliminary model has been created in the same way, as described for our proposed model of the hammerhead ribozyme shown in Fig. 9A, by using the submittal shape of the RNA hammerhead ribozyme (PDB-ID: 2GOZ). Two hybrid double helices (DH1 and DH2), each consisting of one RNA and one DNA strand, have been created as standard B-form DNA helices by using our ERNA-3D software. Subsequently, these two helices have been superimposed onto the corresponding stems of the submittal hammerhead ribozyme structure. The docking points (P-respectively O3′-atoms) at the ends of these double helices have been positioned close enough to the catalytic centre to ensure the simple connection to both single strands of the conserved catalytic centre. In the same way as described in Figure 9A, we used the spatial shapes of both strands (forming the catalytic centre of the hammerhead ribozyme) to substitute them with the corresponding DNA nucleotides of our proposed DNAzyme model. This nucleotide substitution has very easily been performed by using the Copy-Structural-Motif function (substitution of nucleotides with nucleotides with different bases, but at same positions and with same orientations) of our ERNA-3D software. The remainder of the DNA enzymatic loop, the nucleotides T14 to A18 (nucleotide numbering of our proposed model) have also been substituted with the nucleotides of the RNA double helix of the submittal hammerhead ribozyme, but due to the fact, that in this case the DNA loop does not form a real double helix, both strands have been pulled away a little bit by using the interactive chain translation method of our ERNA-3D software. In this way, we obtained a shape for the DNA enzymatic loop, which is similar to an RNA double helix, but which does not form canonical Watson–Crick base-pairs. At last, the L-form of this molecule has simply been created by the negating of the z-components of its atomic coordinates [26].

From our three-dimensional modeling experiments we can conclude that the sequence specific cleavages of LRNA2 by the L-hammerhead ribozyme and L-DNAzyme are feasible at the cleavage points observed in our experiments.

### Concluding Remarks

The results obtained in this study demonstrate for the first time that L-hammerhead ribozymes and L-DNAzymes are capable of cleaving L-RNA target sequences specifically and very efficiently. Stability studies performed for the L-hammerhead ribozyme and L-DNAzyme with three different human sera demonstrated that these two L-RNA cleaving enzymes are very stable in different sera even up to the 72 hours tested. This observation is in agreement with our earlier results obtained with Spiegelmers [3], [4]. The reason for this stability lies in the fact that in nature no nucleic acids exist in the L-form, just in their D-form. Thus, there have been no reasons during the evolution to synthesize any enzymes degrading L-form nucleic acids. The stability of the Spiegelzymes were further documented by incubation of an L-RNA target with the commercially obtained ribonucleases T1, V1, S1, and T2.

The fact that the L-hammerhead ribozyme could very efficiently cleave its L-RNA target, in a human serum, indicates that the mirror image ribozymes may be suitable for employment in biological environments.

Considering the efficient activities of the L-hammerhead ribozyme and L-DNAzyme to hydrolyze sequence specifically L-RNA sequences, they may be well suited as an antidote against several Spiegelmers, which are currently in second medical testing phases by the Noxxon Pharma AG. Not only that, it can also be envisioned that new kinds of bio chips can be developed on the Spiegelmer Spiegelzyme basis for diagnostic purposes and the isolation of specific proteins. Also, the construction of new kind of nanoparticles should be possible, based upon a combination of Spiegelmers and Spiegelzymes for the site directed delivery of drugs.

Intriguing will also be to determine the mechanism how these mirror image enzymes are functioning and compare those with the mechanism determined for the natural hammerhead ribozymes. Here it will be especially interesting, if one could compare the L-hammerhead ribozyme mechanism with that determined by Scott and collaborators [21], [34] for the natural D-form hammerhead ribozyme. Since the determination of the three-dimensional structure at high resolution requires suitable crystals, we are currently working on the crystallization of the L-hammerhead ribozyme and the L-DNAzyme. In the meantime we have turned to three-dimensional model building of the two Spiegelzymes used in this study. Indeed, we are able to present with this study for the first time structural models, not only of L-nucleic acids, but for both Spiegelzymes as they are binding and hydrolyzing their L-RNA targets.

In summary, with the first description of functionally active L-hammerhead ribozymes and L-DNAzymes, which were shown to specifically hydrolyze L-RNA targets the principles are laid down that these molecules may become important tools for future applications in biotechnology and molecular medicine. These theoretical potentials are further supported by the very high stabilities of these enzymes in human sera, and the recent evidence that mirror image nucleic acids are not toxic and not immunogenic.
